# How long do revised and multiply revised hip replacements last? A retrospective observational study of the National Joint Registry

**DOI:** 10.1016/S2665-9913(22)00097-2

**Published:** 2022-06-23

**Authors:** Kevin Deere, Michael R Whitehouse, Setor K Kunutsor, Adrian Sayers, James Mason, Ashley W Blom

**Affiliations:** a Musculoskeletal Research Unit, Translational Health Sciences, Bristol Medical School, Southmead Hospital, Bristol, UK; b National Institute for Health Research Bristol Biomedical Research Centre, University Hospitals Bristol and Weston NHS Foundation Trust and University of Bristol, Bristol, UK; c Warwick Clinical Trials Unit, Warwick Medical School, University of Warwick, Coventry, UK

## Abstract

**Background:**

Hip replacements are common and effective operations but patients that undergo this intervention are at risk of the replacements failing, requiring costly and often complex revision surgery with poorer outcomes than primary surgery. There is paucity of reliable data examining the treatment pathway for hip replacements over the life of the patient in terms of risk of revision and re-revisions. We aim to provide detailed information on the longevity of hip revision surgery.

**Methods:**

We did a retrospective observational registry-based study of the National Joint Registry, using data on hip replacements from all participating hospitals in England and Wales, UK. We included data on all first revisions, with an identifiable primary procedure, with osteoarthritis as the sole indication for the original primary procedure. Kaplan-Meier estimates were used to determine the cumulative probability of revision and subsequent re-revision after primary hip replacement. Analyses were stratified by age and gender, and the influence of time from first to second revision on the risk of further revision was explored.

**Findings:**

Between April 1, 2003, and Dec 31, 2019, there were 29 010 revision hip replacements with a linked primary episode. Revision rates of revision hip replacements were higher in patients younger than 55 years than in older age groups. After revision of primary total hip replacement, 21·3% (95% CI 18·6–24·4) of first revisions were revised again within 15 years, 22·3% (20·3–24·4) of second revisions were revised again within 7 years, and 22·3% (18·3–27·0) of third revisions were revised again within 3 years. After revision of hip resurfacing, 23·7% (95% CI 19·6–28·5) of these revisions were revised again within 15 years, 21·0% (17·0–25·8) of second revisions were revised again within 7 years, and 19·3% (11·9–30·4) of third revisions were revised again within 3 years. A shorter time between revision episodes was associated with earlier subsequent revision.

**Interpretation:**

Younger patients are at an increased risk of multiple revisions. Patients who undergo a revision have a steadily increasing risk of further revision the more procedures they undergo, and each subsequent revision lasts for approximately half the time of the previous one. Although hip replacements are effective for improving pain and function and usually last a remarkably long time, if they are revised, successive revisions are progressively and markedly less successful.

**Funding:**

NIHR Biomedical Research Centre at the University Hospitals Bristol and Weston NHS Foundation Trust and the University of Bristol, Healthcare Quality Improvement Partnership; and the National Joint Registry.

## Introduction

Hip replacements are a common operation and an extremely effective treatment for improving pain and disability for most patients with advanced hip joint disease such as osteoarthritis.^1^ Hip replacements are cost-effective interventions and have high patient satisfaction ratings.^2^ The lifetime risk at age 50 years of undergoing hip replacement is approximately 11% for women and 7% for men.^3^ Over 100 000 primary total hip replacements are done annually in England and Wales.^4^ Rates of primary hip replacement have been predicted to increase by 129% between 2014 and 2030 in the USA,^5^ over 200% in Australia between 2019 and 2030,^6^ 134% in England and Wales between 2012 and 2030,^7^ and 29% in Germany between 2016 and 2040.^8^

In 2019, a meta-analysis^9^ found that 57·9% of total hip replacements last over 25 years. Despite high overall success rates, some hip replacements require revision surgery, which is associated with higher costs and worse outcomes than the primary surgery.^10^, ^11^ The 2021 data from the National Joint Registry in England and Wales, shows that, on average, 4·3% of primary hip replacements are revised within 10 years, which is comparable with the 4·1% of primary knee replacements that are revised within 10 years.^4^ Hip replacements are most commonly revised for aseptic loosening (prosthesis-time incidence rate [PTIR] per 1000 years: 1·32), dislocation or subluxation (0·80), periprosthetic fracture (0·70), adverse reactions to particulate debris (0·70), and infection (0·69).^4^ Dislocation or subluxation is the most common cause of revision within a year of the primary procedure (2·47), becoming a less common cause the longer a hip is in situ (0·42 at 5–7 years after the primary procedure). By contrast, adverse reactions to particulate debris is uncommon in the early period after a primary hip replacement (0·09 for a revision within a year), becoming more prominent the longer the hip replacement is in situ (eg, 1·16 at 5–7 years since primary operation).^4^ The mean age to undergo total hip replacement is 69 years and the lifetime risk of revision for patients aged 65–69 years is approximately 15% for men and 10% for women.^12^ Hip resurfacing has markedly higher revision rates (14·8% at 17 years) than conventional uncemented (8·8% at 17 years), and conventional cemented (6·1% at 17 years) hip replacements.^4^ These differences are partially due to selection effects as resurfacings are more commonly used in younger male patients; however, revision rates are still significantly higher when accounting for these differences.^13^ Many hip replacements, including hip resurfacings, will therefore be revised in the lifetime of the recipient, resulting in over 120 000 revision hip replacements being done in England and Wales between 2004 and 2020.^4^ Revision hip replacements are more expensive and result in worse function and pain than do primary hip replacements.^10^ A study of over 200 000 revision hip replacements in the USA revealed average costs of US$87 000 per revision done in 2014.^14^

**Research into context**

**Evidence before this study**

Total hip replacement is commonly done globally for pain and disability associated with end-stage osteoarthritis. It is a highly successful and cost-effective intervention with majority of implants lasting over 25 years. However, some patients experience complications which might result in revision surgery. Several studies have reported that revision hip replacements are not as effective as the primary surgery, are associated with worse outcomes and are costly to health-care systems. With the ageing population, the demand for total hip replacements will increase substantially, which will be accompanied by a corresponding increase in revision surgeries. Therefore, it will be useful to know long first revisions and their subsequent re-revisions last primary hip replacement. This data will be valuable for planning health-care provision and help patients to make informed choices. We searched MEDLINE and Embase from inception to Oct 10, 2021, for long-term observational cohort studies and meta-analyses that have reported on the risk of revision and subsequent re-revision following primary hip replacement or resurfacing. We searched using the terms: “hip replacement”, “hip arthroplasty”, “THA”, “THR”, “hip resurfacing”, which were related to the population; and “revision”, “re-revision”, “reoperation”, “survival”, “failure”, which were related to outcomes. We identified several relevant studies with small sample sizes that had retrospectively evaluated patients with first-time revised total hip replacements or resurfacings and their subsequent risk of re-revision. One study based on 69 primary total hip replacements reported 13 first revisions during the average 11·5 years follow-up, but none of these 13 revisions needed a re-revision within 10 years after re-implantation. One recently reported a detailed breakdown of how long first revisions and their subsequent re-revisions last after the primary knee replacement, however no study has evaluated this for hip joints.

**Added value of this study**

To our knowledge, this is the first study to describe the treatment pathways of patients after primary hip replacement and resurfacing with osteoarthritis as the sole reason for the procedure. Using data from the largest joint registry in the world, Kaplan-Meier estimates were used to describe the cumulative probability of revision hip replacement in all cases where a link was identified between a known primary stemmed hip replacement or resurfacing and subsequent revisions. A total of 29 010 first documented revision hip replacements were linked with the primary surgery. Of 25 082 first revision total hip replacements, 21% were re-revised within 15 years, 22% of second revisions were revised again within 7 years, and 22% of third revisions revised within 3 years. The revision trend was similar for 3928 first revised resurfaced hips. For both study populations, the risk of multiple revisions was similar in males and females, but higher in younger patients (<55 years).

**Implications of all the available evidence**

Hip replacements are very effective for the treatment of osteoarthritis and they usually last a long time. However, if they are revised, subsequent replacements become less and less successful. To reduce the adverse effect of revision surgery on patients and the health-care system, optimal prosthetic implants with low failure rates should be employed for hip surgery, especially in patients younger than 55 years who are at substantial risk of having multiple revisions. Furthermore, patients need to be counselled appropriately regarding the complex journey involved in a hip replacement to allow them to make informed decisions.

For patients to make informed choices they need to understand the entire patient pathway from intervention to death. To our knowledge, no study has specifically evaluated the risk of subsequent re-revisions after the first and second revisions after primary hip replacement, although we have previously studied this after primary knee replacement.^15^ Using data from the largest joint replacement database in the world we aimed to ascertain how long revision hip replacements last and how long each subsequent revision lasts before having repeat revision.

## Methods

### Data source

In this retrospective observational registry-based study we analysed longitudinal data from the National Joint Registry, which covers England, Wales, Northern Ireland, the Isle of Man, and the States of Guernsey; collects information on hip, knee, ankle, elbow, and shoulder joint replacement surgery; and monitors the performance of joint replacement implants. We collected data on hip replacement revision procedures gathered in the National Joint Registry from hospitals in England and Wales, since its inception in April 1, 2003 until Dec 31, 2019. A data quality audit of the National Joint Registry in 2017–18 showed over 95% capture of primary hip data and approximately 91% capture of revision hip replacements.^16^ Further details on the specifics of the National Joint Registry data capture and the Minimum Data Set forms can be viewed on the National Joint Registry website.^17^ Approval for this study was granted by the National Joint Registry Research sub-committee. Written consent was granted by patients for inclusion of their data and its use in research within the National Joint Registry.

### Procedures and outcomes

The National Joint Registry hip data was initially prepared for this analysis in the same manner as described in the 2020 National Joint Registry annual report.^16^ Briefly, the data were cleaned by removing: records with missing information, duplicate procedures, and records where we were unable to ascertain a logical sequence of primary and revision procedures. Additionally, we excluded procedures with an unknown primary hip type, and primary procedures that had an indication other than osteoarthritis. Primary procedures are first time hip replacements. Therefore, our inclusion criteria were all first revisions, with an identifiable primary procedure, with osteoarthritis as the sole indication for the original primary procedure. From this we split our remaining population into two distinct groups: firstly, all total hips with osteoarthritis as the sole indication for the primary procedure, and secondly, all hip resurfacing primaries with osteoarthritis as the sole indication for the primary.

The National Joint Registry data contained details of the operation date and procedure type. Revisions in the National Joint Registry are defined as any procedure where an implant is added, modified, or removed. Revision procedures in the National Joint Registry are classified as single-stage (where the complete revision procedure is done during one surgical episode), stage one of two-stage, or stage two of two-stage revisions (where a complete revision procedure is done during at least two separate surgical episodes, on different dates with the revision episode starting with the stage one procedure). Debridement and implant retention (DAIR) with or without modular exchange were included as single-stage procedures. From these procedures we derived our revision episodes. We assumed that a single stage procedure was a distinct episode, as was a DAIR. In the case of two-stage revision, where a second stage revision was recorded within 365 days of a first stage revision procedure, we considered this to be a single distinct revision episode. This classification allowed multiple stage one of two-stage procedures to occur before triggering a new episode, as long as the complete first record of a first stage revision procedure was followed by a second stage revision procedure within 365 days. From this we could ascertain the date of the first revision (second prosthetic hip) and the dates of the beginning of any subsequent revision episodes potentially forming a second, third, and fourth revision episode (third, fourth, and fifth prosthetic hips).

The National Joint Registry data also contained details on gender, age at procedure, and the American Society of Anesthesiologists (ASA) grade at the time of the procedure. Date of death was provided by the Office of National Statistics, if the National Health Service number was traceable, and was linked to the National Joint Registry data before our receipt.

We also derived a variable to describe the time interval between the previous two episodes to determine how long the prosthetic hip was in situ before revision. This was categorised into less than 1 year, between 1 and 3 years, between 3 and 5 years, and greater than 5 years.

### Statistical analysis

We used Kaplan-Meier estimates to describe the cumulative probability of revision hip replacement in all cases where we could find a link between a known primary operation and subsequent revision of the same side hip (left or right). Linking to an associated primary operation allowed us to be sure of the sequence of events and therefore correctly identify the first revision procedure. Any revision procedures without a linked primary were excluded from the analysis as we were not able to ascertain their place in the chain of events. All data were censored at date of death or at the end of the study period (Dec 31, 2019). There were three parts to our analysis in both study populations (total hips and resurfacings): Kaplan-Meier estimates of the survivorship of the first revision, the second revision (ie, the re-revision) and the third revision (ie, the second re-revision). For each of these strands we produced Kaplan-Meier estimates with 95% CI, stratified by age (grouped as younger than 55 years, 55 to 64 years , 65 to 74 years, and 75 years and older) and gender. We also calculated the Kaplan-Meier estimates of the survivorship of the first revision stratified by the time interval between the primary operation and the first revision, and the survivorship of the second revision stratified by the time interval between first and second revision.

Kaplan-Meier estimates and 95% CI can become less reliable when the numbers at risk become low. Therefore, consistent with the reporting in the National Joint Registry annual reports, we have highlighted in the tables any estimate where there are less than 250 procedures at risk at any given time point. All analyses were done using Stata/SE (version 15.1).

### Role of the funding source

The funder of the study had no role in study design, data collection, data analysis, data interpretation, or writing of the report.

## Results

**Figure 1:**
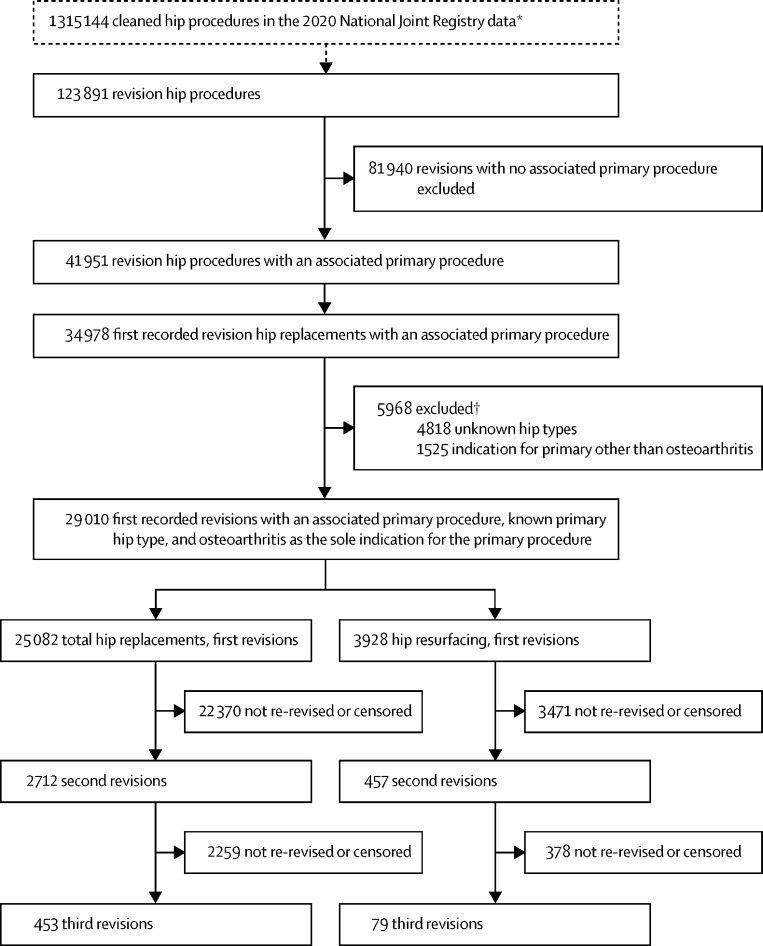
Patient population *1 446 433 hip procedures were recorded in the National Joint Registry. In preparation for the National Joint Registry 2020 annual report (before this study) the data were cleaned by removing 131 289 records with missing information, data entry errors, duplicate records, and removing records in which a logical sequence of revision procedures could not be ascertained. †Counts not mutually exclusive.

The 2020 National Joint Registry annual report cohort consisted of 1 315 144 hip procedures, of which there were 1 191 253 (90·6%) primary hip replacements. (figure 1). In this cohort 1 090 244 (91·5%) of the primary procedures listed osteoarthritis as an indication for surgery, with 1 052 601 (88·4%) stating osteoarthritis as the sole indication for the primary procedure. During cleaning of the National Joint Registry data, before this study,^16^ a small number of observations were lost due to missing or unverifiable data (139 [0·01%] of 1 191 253), thus loss to follow-up is assumed to be negligible.

Between April 1, 2003, and the end of Dec 31, 2019, the National Joint Registry recorded a total of 123 891 revision hip procedures (figure 1). This was the starting point of data cleaning in this analysis. There were 81 940 (66·1%) revision procedures with no linked primary procedure in the data, likely owing to the primary procedure occurring before the National Joint Registry started data collection. We identified 34 978 (28·2%) first documented revisions, with a linked primary procedure in the data. Of these, 5968 (17·1%) had an unknown hip type at primary replacement, or listed an indication for primary replacement other than osteoarthritis, and were thus excluded from the analysis. This gave us 29 010 (23·4%) first linked revisions in our analysis, of which 25 082 (86·5%) were after total hip replacements and 3928 (13·5%) were after hip resurfacings.

Out of the revisions of the total hip population with a linked primary, 2712 (10·8%) of the first revisions were subsequently re-revised (second revision), with 453 (1·8%) going on to have a third revision. In the hip resurfacing population, 457 (11·6%) of the first revisions were subsequently re-revised (second revision), with 79 (2·0%) going on to have a third revision.

Table 1 shows the demographics of patients in the cohort as well as the types of revision they had. The total hip replacement group were on average 10 years older than the resurfacing population at time of first revision (mean 70·2 years [SD 10·6] *vs* 59·8 [9·0]). Revisions in the resurfacing group were more likely to be single stage procedures than in the total hip group, as were second revisions and third revisions. In both study populations the proportion of single stage procedures were reduced in second revisions, and in third revisions, which is probably a reflection of the increased complexity and increased likelihood of undergoing a two-stage revision, typically done for infection, during subsequent revisions. There were incomplete two-stage episodes among the first, second, and third revisions, where either a stage one of two procedure was not followed by a stage two of two within 365 days, or where there was a stage two of two procedure that was not preceded by a stage one of two procedure in the prior 365 days (table 1). We also found 89 observations (0·3% of those with a first revision) where there were multiple stage one of two procedures before a stage two of two procedure. The median time between the first stage one of two and the first occurrence of a stage two of two was 111 days (IQR 73–177).

**Table 1:** Patient characteristics

		**First revision**			**Second revision**			**Third revisions**		
		Male	Female	All	Male	Female	All	Male	Female	All
**Total**										
Total hip replacement		11066 (44·1%)	14016 (55·9%)	25082 (100%)	1215 (44·8%)	1497 (55·2%)	2712 (100%)	207 (45·7%)	246 (54·3%)	453 (100%)
Hip resurfacing		1999 (50·9%)	1929 (49·1%)	3928 (100%)	230 (50·3%)	227 (49·7%)	457 (100%)	36 (45·6%)	43 (54·4%)	79 (100%)
**Mean age, years**										
Total hip replacement		69·6 (10·8)	70·7 (10·5)	70·2 (10·6)	69·2 (10·4)	70·2 (10·2)	69·7 (10·3)	69·3 (9·6)	69·6 (9·9)	69·5 (9·8)
Resurfacing		60·2 (9·2)	59·4 (8·8)	59·8 (9·0)	60·9 (9·7)	59·3 (9·4)	60·1 (9·6)	62·3 (8·8)	59·6 (7·1)	60·8 (8·0)
**Age group, years**										
<55										
	Total hip replacement	1601 (14·5%)	1767 (12·6%)	3368 (13·4%)	196 (16·1%)	227 (15·2%)	423 (15·6%)	35 (16·9%)	48 (19·5%)	83 (18·3%)
	Resurfacing	925 (46·3%)	1006 (52·2%)	1931 (49·2%)	109 (47·4%)	129 (56·8%)	238 (52·1%)	15 (41·7%)	28 (65·1%)	43 (54·4%)
55–64										
	Total hip replacement	3269 (29·5%)	3858 (27·5%)	7127 (28·4%)	382 (31·4%)	437 (29·2%)	819 (30·2%)	74 (35·7%)	73 (29·7%)	147 (32·5%)
	Resurfacing	848 (42·4%)	810 (42·0%)	1658 (42·2%)	97 (42·2%)	85 (37·4%)	182 (39·8%)	16 (44·4%)	13 (30·2%)	29 (36·7%)
65–74										
	Total hip replacement	3970 (35·9%)	5169 (36·9%)	9139 (36·4%)	425 (35·0%)	560 (37·4%)	985 (36·3%)	72 (34·8%)	92 (37·4%)	164 (36·2%)
	Resurfacing	216 (10·8%)	111 (5·8%)	327 (8·3%)	24 (10·4%)	13 (5·7%)	37 (8·1%)	5 (13·9%)	2 (4·7%)	7 (8·9%)
>75										
	Total hip replacement	2226 (20·1%)	3222 (23·0%)	5448 (21·7%)	212 (17·4%)	273 (18·2%)	485 (17·9%)	26 (12·6%)	33 (13·4%)	59 (13·0%)
	Resurfacing	10 (0·5%)	2 (0·1%)	12 (0·3%)	0	0	0	0	0	0
**ASA grade**										
Grade 1										
	Total hip replacement	984 (8·9%)	1141 (8·1%)	2125 (8·5%)	76 (6·3%)	83 (5·5%)	159 (5·9%)	8 (3·9%)	15 (6·1%)	23 (5·1%)
	Resurfacing	533 (26·7%)	450 (23·3%)	983 (25·0%)	45 (19·6%)	37 (16·3%)	82 (17·9%)	8 (22·2%)	8 (18·6%)	16 (20·3%)
Grade 2										
	Total hip replacement	6669 (60·3%)	8916 (63·6%)	15585 (62·1%)	687 (56·5%)	861 (57·5)	1548 (57·1%)	112 (54·1%)	133 (54·1%)	245 (54·1%)
	Resurfacing	1250 (62·5%)	1319 (68·4%)	2569 (65·4%)	147 (63·9%)	158 (69·6%)	305 (66·7%)	22 (61·1%)	26 (60·5%)	48 (60·8%)
Grade 3										
	Total hip replacement	3174 (28·7%)	3711 (26·5%)	6885 (27·4%)	427 (35·1%)	521 (34·8%)	948 (35·0%)	84 (40·6%)	93 (37·8%)	177 (39·1%)
	Resurfacing	209 (10·5%)	156 (8·1%)	365 (9·3%)	38 (16·5%)	32 (14·1%)	70 (15·3%)	6 (16·7%)	9 (20·9%)	15 (19·0%)
Grade 4										
	Total hip replacement	237 (2·1%)	245 (1·7%)	482 (1·9%)	25 (2·1%)	32 (2·1%)	57 (2·1%)	3 (1·4%)	5 (2·0%)	8 (1·8%)
	Resurfacing	6 (0·3%)	4 (0·2%)	10 (0·3%)	0	0	0	0	0	0
Grade 5										
	Total hip replacement	2 (<0·1%)	3 (<0·1)	5 (<0·1)	0	0	0	0	0	0
	Resurfacing	1 (0·1%)	0	1 (<0·1%)	0	0	0	0	0	0
**Previous hip in-situ**										
<1 year										
	Total hip replacement	3220 (29·1%)	3772 (26·9%)	6992 (27·9%)	599 (49·3%)	726 (48·5%)	1325 (48·9%)	104 (50·2%)	129 (52·4%)	233 (51·4%)
	Resurfacing	304 (15·2%)	127 (6·6%)	431 (11·0%)	68 (29·6%)	56 (24·7%)	124 (27·1%)	17 (47·2%)	14 (32·6%)	31 (39·2%)
1–3 years										
	Total hip replacement	2332 (21·1%)	2837 (20·2%)	5169 (20·6%)	353 (29·1%)	455 (30·4%)	808 (29·8%)	55 (26·6%)	79 (32·1%)	134 (29·6%)
	Resurfacing	311 (15·6%)	289 (15·0%)	600 (15·3%)	67 (29·1%)	64 (28·2%)	131 (28·7%)	10 (27·8%)	18 (41·9%)	28 (35·4%)
3–5 years										
	Total hip replacement	1582 (14·3%)	2193 (15·6)	3775 (15·1%)	144 (11·9%)	156 (10·4%)	300 (11·1%)	31 (15·0%)	24 (9·8%)	55 (12·1%)
	Resurfacing	372 (18·6%)	370 (19·2)	742 (18·9%)	32 (13·9%)	48 (21·1%)	80 (17·5%)	4 (11·1%)	7 (16·3%)	11 (13·9%)
≥5 years										
	Total hip replacement	3932 (35·5%)	5214 (37·2%)	9146 (36·5%)	119 (9·8%)	160 (10·7%)	279 (10·3%)	17 (8·2%)	14 (5·7%)	31 (6·8%)
	Resurfacing	1012 (50·6%)	1143 (59·3%)	2155 (54·9%)	63 (27·4%)	59 (26·0%)	122 (26·7%)	5 (13·9%)	4 (9·3%)	9 (11·4%)
**Revision procedure**										
DAIR										
	Total hip replacement	149 (1·3%)	131 (0·9%)	280 (1·1%)	15 (1·2%)	25 (1·7%)	40 (1·5%)	9 (4·3%)	3 (1·2%)	12 (2·6%)
	Resurfacing	0	0	0	0	1 (0·4%)	1 (0·2%)	0	0	0
Excision										
	Total hip replacement	50 (0·5%)	57 (0·4%)	107 (0·4%)	11 (0·9%)	32 (2·1%)	43 (1·6%)	2 (1·0%)	4 (1·6%)	6 (1·3%)
	Resurfacing	2 (0·1%)	5 (0·3%)	7 (0·2%)	0	1 (0·4%)	1 (0·2%)	0	1 (2·3%)	1 (1·3%)
Single stage										
	Total hip replacement	9378 (84·7%)	12569 (89·7%)	21947 (87·5%)	789 (64·9%)	1112 (74·3%)	1901 (70·1%)	116 (56·0%)	172 (69·9%)	288 (63·6%)
	Resurfacing	1872 (93·6%)	1847 (95·7%)	3719 (94·7%)	193 (83·9%)	197 (86·8%)	390 (85·3%)	25 (69·4%)	32 (74·4%)	57 (72·2%)
Complete two-stage episode										
	Total hip replacement	665 (6·0%)	556 (4·0%)	1221 (4·9%)	155 (12·8)	113 (7·5%)	268 (9·9%)	35 (16·9%)	25 (10·2%)	60 (13·2%)
	Resurfacing	48 (2·4%)	24 (1·2%)	72 (1·8%)	11 (4·8%)	8 (3·5%)	19 (4·2%)	3 (8·3%)	4 (9·3%)	7 (8·9%)
Stage one of two episode*										
	Total hip replacement	458 (4·1%)	382 (2·7%)	840 (3·3%)	108 (8·9%)	88 (5·9%)	196 (7·2%)	21 (10·1%)	18 (7·3%)	39 (8·6%)
	Resurfacing	25 (1·3%)	23 (1·2%)	48 (1·2%)	12 (5·2%)	9 (4·0%)	21 (4·6%)	3 (8·3%)	3 (7·0%)	6 (7·6%)
Stage two of two episode†										
	Total hip replacement	366 (3·3%)	320 (2·3%)	686 (2·7%)	137 (11·3%)	127 (8·5%)	264 (9·7%)	24 (11·6%)	24 (9·8%)	48 (10·6%)
	Resurfacing	52 (2·6%)	30 (1·6%)	82 (2·1%)	14 (6·1%)	11 (4·8%)	25 (5·5%)	5 (13·9%)	3 (7·0%)	8 (10·1%)

Data are n (%) or mean (SD). ASA=American Society of Anesthesiologists. DAIR=Debridement and implant retention.

* Stage one of two is the first stage of a revision procedure usually done as two operations (ie, under two separate anaesthetics). Here, we present stage one of two procedures not followed by a stage 2 of 2 procedure within 365 days.

† Stage two of two is the second stage of a revision procedure done as two operations. Here, we present counts of stage two of two procedures not preceded by a stage one of two in the prior 365 days.

The comorbidity profile of patients also progressively worsened in both study populations. In the total hip replacement group, 17 710 (70·6%) of 25 082 patients with first time revisions were classified as ASA grade 1 or 2, compared with 1707 (63·0%) of 2712 second revisions, and 268 (59·2%) of 453 third revisions. There was a similar trend of worsening comorbidity in the resurfacing population with 3552 (90·4%) of 3928 patients with first time revisions classified as ASA grade 1 or 2, compared with 387 (84·6%) of 457 second revisions, and 64 (81·0%) of 79 third revisions. Amongst total hip replacement first revisions there were 3589 deaths (14·3%) during the follow-up period, compared with 382 (14·1%) of second revisions, and 49 (10·8%) of third revisions. In the resurfacing population there were 176 deaths (4·5%) during the follow-up period of the first revision, compared with 14 (3·1%) in the second revision, and four (5·1%) in the third revision.

**Figure 2:**
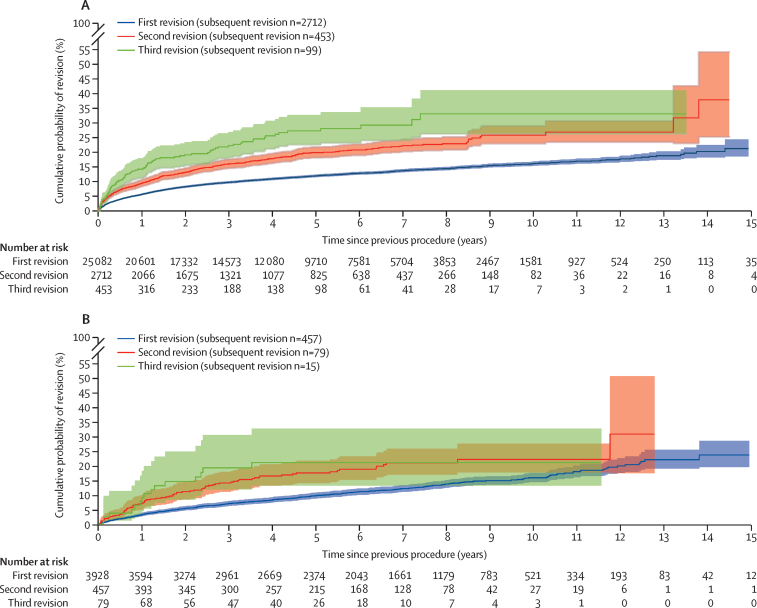
Kaplan-Meier estimates of cumulative revision in patients with linked primary hip replacements by first revision, second revision, and third revision (A) Total hip replacements. (B) Resurfacing. Populations not mutually exclusive; second and third revision are a subset of the first revision population.

Figure 2 shows that 21·3% (95% CI 18·6–24·4) of first revisions (second prosthetic hip) after initial primary total hip replacement were revised (to third prosthetic hips) within 15 years. Whereas 22·3% (20·3–24·4) of second revisions (third prosthetic hips) were revised (to fourth prosthetic hips) within 7 years, and 22·3% (18·3–27·0) of third revision (fourth prosthetic hip) were revised (to fifth prosthetic hips) within 3 years.

Figure 2 shows a similar trend in the resurfacing study population. 23·7% (95% CI 19·6–28·5) of first revisions (second prosthetic hip) after initial hip resurfacing were revised within 15 years; 21·0% (17·0–25·8) of second revisions (third prosthetic hips) of resurfacings were revised within 7 years, whereas 19·3% (11·9–30·4) of third revisions (fourth prosthetic hip) were revised within 3 years. Data pertaining to reasons for the revisions in first, second, and third revisions can be viewed in the appendix (pp 2,3).

Survivorship of the first revision was similar in males and females for both study populations as shown in table 2. A similar pattern was seen for males and females in revision rates for second and third revisions (appendix p 1 for survivorship of second revision table; third revision data not shown). Re-revision was higher in younger patients, in both males and females (table 2). In females younger than 55 years the re-revision rate of total hip replacements was 21·6% (95% CI 18·1–25·5) at 10 years, compared with 15·7% (14·2–17·3) in females aged 65 to 74 years. A similar pattern was shown in the resurfacing group. For example, in males younger than 55 years the re-revision rate was 16·8% (13·7–20·6) at 7 years, compared with 10·4% (8·3–9·0) in males aged 55 to 64 years. It should be noted that the sample size within the different age groups decreased to below 250 by 10 years of follow-up, and as such these estimates should be interpreted with caution.

**Table 2:** Kaplan-Meier estimates of cumulative re-revision in both study populations, stratified by sex and age

	**Revision (%)**	**Time since first revision**						
		1 year	3 years	5 years	7 years	10 years	13 years	15 years
**All cases**								
Total hip replacement	25082 (100%)	5·6 (5·3–5·9)	9·7 (9·3–10·1)	11·9 (11·5–12·4)	13·7 (13·2–14·2)	16·1 (15·4–16·8)	18·9 (17·5–20·4)	21·3 (18·6–24·4)*
Resurfacing	3928 (100%)	3·3 (2·7–3·9)	7·0 (6·3–7·9)	9·8 (8·8–10·8)	12·2 (11·1–13·4)	15·9 (14·4–17·6)	22·1 (19·0–25·4)*	23·7 (19·6–28·5)*
**Females, all ages**								
Total hip replacement	14016 (55·9%)	5·5 (5·1–5·8)	9·6 (9·1–10·1)	11·6 (11·0–12·2)	13·4 (12·7–14·1)	15·6 (14·7–16·6)	19·2 (17·2–21·5)*	20·8 (18·0–24·1)*
Resurfacing	1929 (49·1%)	3·0 (2·4–3·9)	6·8 (5·7–8·1)	9·9 (8·6–11·4)	12·4 (10·8–14·2)	15·6 (13·5–17·9)*	21·2 (16·9–26·5)*	25·2 (17·6–35·3)*
**Female, aged <55 years**								
Total hip replacement	1017 (4·1%)	6·8 (5·4–8·5)	11·7 (9·8–13·9)	15·2 (13·0–17·8)	17·7 (15·2–20·6)	21·6 (18·1–25·5)*	26·3 (21·0–32·7)*	26·3 (21·0–32·7)*
Resurfacing	513 (13·1%)	3·9 (2·5–6·0)	8·7 (6·6–11·6)	14·4 (11·6–17·9)	17·2 (14·1–21·0)	20·8 (17·0–25·2)*	27·1 (20·3–35·5)*	
**Female, aged 55 to 64 years**								
Total hip replacement	2522 (10·1%)	5·3 (4·5–6·3)	11·1 (9·9–12·5)	13·3 (12·0–14·9)	15·0 (13·5–16·6)	17·4 (15·5–19·4)	23·0 (18·4–28·6)*	25·1 (19·4–32·1)
Resurfacing	875 (22·3%)	3·2 (2·2–4·6)	6·0 (4·6–7·9)	8·3 (6·6–10·5)	10·8 (8·7–13·3)	13·1 (10·6–16·1)*	18·9 (13·5–26·0)*	23·9 (14·7–37·4)*
**Female, aged 65 to 74 years**								
Total hip replacement	5112 (20·4%)	5·3 (4·7–5·9)	9·8 (9·0–10·7)	11·9 (10·9–12·9)	13·5 (12·4–14·6)	15·7 (14·2–17·3)	17·2 (14·9–19·9)*	19·2 (15·1–24·3)*
Resurfacing	481 (12·2%)	2·1 (1·2–4·0)	6·5 (4·5–9·3)	7·8 (5·5–10·9)*	9·5 (6·8–13·2)*	16·1 (9·6–26·3)*	16·1 (9·6–26·3)*	
**Female, aged ≥75 years**								
Total hip replacement	5365 (21·4%)	5·4 (4·8–6·1)	8·0 (7·2–8·8)	9·3 (8·5–10·3)	11·3 (10·2)	12·4 (11·0–14·1)*	15·6 (12·3–19·6)*	15·6 (12·3–19·6)*
Resurfacing	60 (1·5%)	0	2·6 (0·4–17·3)*	2·6 (0·4–17·3)*	2·6 (0·4–17·3)*			
**Males, all ages**								
Total hip replacement	11066 (44·1%)	5·8 (5·3–6·2)	9·9 (9·3–10·6)	12·3 (11·7–13·1)	14·1 (13·3–14·9)	16·8 (15·6–17·9)	18·4 (16·7–20·2)*	22·4 (17·0–29·2)*
Resurfacing	1999 (50·9%)	3·5 (2·8–4·4)	7·3 (6·2–8·6)	9·6 (8·3–11·1)	12·0 (10·4–13·7)	16·2 (14·0–18·6)	22·7 (18·6–27·6)*	22·7 (18·6–27·6)*
**Male, aged <55 old**								
Total hip replacement	977 (3·9%)	5·3 (4·0–6·9)	11·8 (9·9–14·2)	16·2 (13·8–19·0)	18·7 (16·0–21·9)	20·4 (17·3–24·1)*	22·3 (17·9–27·5)*	29·3 (17·9–45·8)*
Resurfacing	539 (13·7%)	3·4 (2·1–5·3)	9·2 (7·0–12·1)	12·7 (10·1–16·1)	16·8 (13·7–20·6)	20·8 (17·0–25·3)*	24·3 (18·7–31·2)*	24·3 (18·7–31·2)*
**Male, aged 55 to 64 years**								
Total hip replacement	2276 (9·1%)	5·6 (4·7–6·6)	10·3 (9·1–11·7)	13·3 (11·8–15·0)	15·6 (13·9–17·5)	18·9 (16·6–21·6)*	21·0 (17·4–25·1)*	27·6 (17·0–42·8)*
Resurfacing	792 (20·2%)	3·6 (2·5–5·2)	6·4 (4·8–8·4)	8·9 (7·0–11·3)	10·4 (8·3–13·0)	14·9 (11·8–18·9)*	24·6 (18·2–32·8)*	24·6 (18·2–32·8)*
**Male, aged 65 to 74 years**								
Total hip replacement	3922 (15·6%)	5·8 (5·1–6·6)	9·9 (8·9–10·9)	12·2 (11·1–13·4)	13·7 (12·4–15·0)	16·4 (14·8)	18·3 (15·8–21·1)*	18·3 (15·8–21·1)*
Resurfacing	571 (14·5%)	3·4 (2·2–5·3)	6·7 (4·8–9·3)	7·3 (5·3–9·9)	9·2 (6·7–12·4)*	11·9 (8·3–17·1)*	13·8 (9·2–20·6)*	13·8 (9·2–20·6)*
**Male, aged ≥75 years**								
Total hip replacement	3891 (15·5%)	5·9 (5·2–6·7)	9·1 (8·1–10·1)	10·2 (9·1–11·4)	11·6 (10·3–13·1)	13·4 (11·4–15·8)*	13·4 (11·4–15·8)*	..
Resurfacing	97 (2·5%)	3·2 (1·0–9·5)*	7·0 (3·2–15·1)*	7·0 (3·2–15·1)*	7·0 (3·2–15·1)*	38·0 (8·6–92·3)*	..	..

Data are N (%) or % (95% CI). Percentages calculated as a percentage of all cases.

* Fewer than 250 cases remained at risk at these timepoints. Cells with “..” indicate that the number at risk is below ten; therefore, estimates have been removed as they would be highly unreliable.

**Figure 3:**
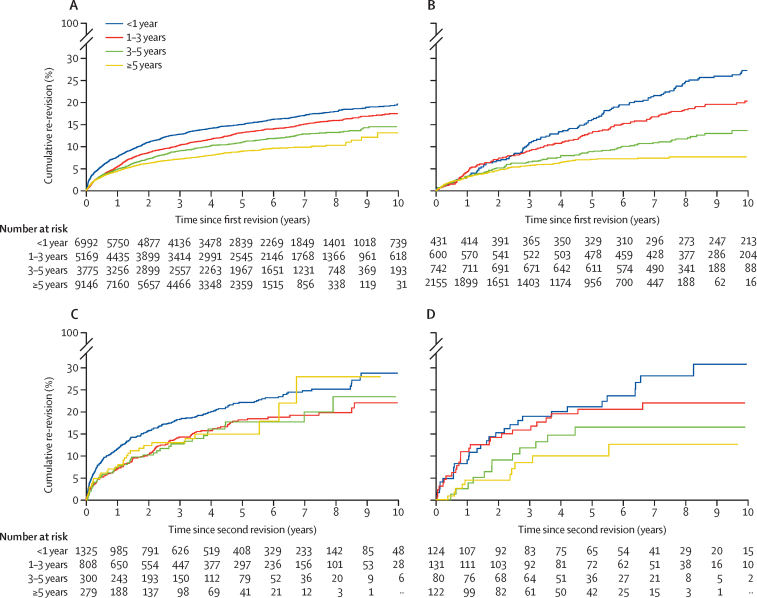
Kaplan-Meier estimates of cumulative probability of revision by time between previous revision episodes, in patients with linked primary hip replacements, stratified by total hip replacements and resurfacing (A) Total hip replacement survivorship of first revision (second prosthetic hip). (B) Resurfacing survivorship of first revision (second prosthetic hip). (C) Total hip replacement survivorship of second revision (third prosthetic hip). (D) Resurfacing survivorship of second revision (third prosthetic hip).

Figure 3 shows the trend between cumulative revision and the time interval between the previous two episodes. Generally, the longer the previous prosthesis was in situ the lower the subsequent revision rate. This is seen in both study populations, for survivorship of the first revision (second prosthetic hip) and the second revision (third prosthetic hip). For example, 7·6% (95% CI 7·0–8·3) of total hip primaries that were revised within 1 year are re-revised (second revision) to a third prosthetic hip within a year and 14·9% (14·0–15·9) within 5 years, whereas total hip primaries that last 5 years or more have only a 4·4% (4·0–4·8) risk of second revision, to a third prosthetic hip, at 1 year and 8·9% (8·2–9·7) at 5 years. The numbers at risk for both plots showing survivorship of third prosthetic hip are low within 1 or 2 years. As such these estimates might be less reliable and should therefore be interpreted with caution.

## Discussion

Previously we have shown that if a patient undergoes a primary total hip replacement, there is approximately a 10% chance that the hip will be revised within 15 years, 30% at 20 years, and 40% at 25 years.^9^ We have also previously shown that revision rates are higher after hip resurfacing than after conventional hip replacement, particularly in women.^13^ In this study, we showed that if the primary hip resurfacing or primary total hip replacement undergoes a first revision to a second prosthetic hip, there is an approximate 20% chance that this will be revised within 15 years requiring a second revision (implantation of a third prosthetic hip). The second revision has an approximate 20% chance of needing a third revision within 7 years (a fourth prosthetic hip) which in turn has an approximate 20% chance of undergoing a fourth revision (fifth prosthetic hip) within 3 years. Furthermore, the longer the primary prosthetic hip lasts, the longer the first revision (second prosthetic hip) is likely to last, although the risk of needing further revision is higher in younger patients.

We present more detailed data than previous reports on the topic. Jafari and colleagues^18^ reported a 17% re-revision rate at 5 years from a leading joint replacement unit in the USA, but it is unclear if these were all first revisions or not. The Norwegian Arthroplasty Register reported a 25% re-revision rate at 10 years after aseptic revisions.^19^ The Australian Orthopaedic Association National Joint Replacement Registry reported a 26% re-revision rate at 10 years following revision after hip resurfacing.^20^ The National Joint Registry annual reports do show a secular improvement in survivorship after both primary hip and knee replacement over the past decade which might account for our results being more favourable than previous reports from elsewhere. Alternatively, case ascertainment might be better in other national registries, although a recent comprehensive data audit done by the National Joint Registry suggests that case ascertainment in our data set is above 90% for revisions.

There is a focus in national quality improvement programmes on ensuring optimisation of the primary procedure to reduce the risk of complications and to minimise costs.^21^ The data we present here reinforces the need for such programmes because of the high burden created by revision hip replacement, particularly if this happens early. The risk of needing to have further revision is substantially higher following revision total hip replacement than it is after a primary total hip replacement. Previous work has also shown that complication rates and readmission rates are higher, and patient reported outcomes are worse after revision hip replacement than primary hip replacement.^10^, ^22^, ^23^ Our data shows that there is a higher proportion of patients requiring two stage revision procedures at subsequent revision episodes reflecting the increased complexity and the requirement for multiple stage procedures which patients prefer to avoid.^24^

Patients and surgeons thus need to understand that even though hip replacements are excellent at improving pain and function^1^ and usually last a remarkably long time, if they are revised, successive replacements (revision procedures) are progressively and markedly less successful. We should thus make every effort to aim for a strategy of one replacement to last a lifetime to optimise patient outcome, reduce the treatment burden on patients, and to reduce the high costs associated with performing revision hip replacements. Detailed work has been done as to which prosthetic combinations for primary joint replacement have a proven track record in different ages in both genders.^16^, ^25^ Joint replacement registers provide an ideal tool to constantly update and expand this information. Decision aid tools can assist in helping patients and clinicians make informed choices around primary joint replacement^26^, ^27^ by highlighting risks to groups at higher risk of poor outcomes, such as men and young patients, as shown here and in previous studies.^28^, ^29^

While planning this analysis we had a choice between using a competing risks model or Kaplan-Meier estimates. We chose a Kaplan-Meier approach as we believe estimates of net failure better describe the failure or replacement of an implant.^30^ Additionally, to aid the use of these results across the world we feel that an estimate which is not conditional on background mortality will be preferable.

In this study we noted that there were numerous incomplete two-stage revisions. Some of these were enforced by the way we dealt with the data: a gap exceeding 365 days between stage one of two and a stage two of two resulted in the stage two of two procedure being classified as a new episode, thus being registered as an incomplete two-stage revision. This was done as we felt it was unlikely, because of the typical time intervals described in the literature between stages, that a planned two stage revision would take place with more than 365 days between the stage one of two and stage two of two revision procedures. However, there are other plausible reasons for an incomplete two-stage revision being recorded. For instance, a patient with an infection that is successfully treated by a stage one of two might decide they do not wish to have a further procedure. In some cases, this could happen when a two-stage revision was planned, in others, it could be as part of a semi-planned procedure where at stage one of two, implants are loosely fixed in place with a plan to only carry out a stage two of two revision if the patient's symptoms require this.^31^ At the inception of the National Joint Registry, it is possible that stage one of two procedures were done before data capture commencing with the stage two of two done after data capture commenced. Similarly, stage one of two revisions might have been done before the administrative censor date but the stage two of two revision were done after this date. It is possible that some stage one of two procedures were done under emergency conditions and a Minimum Data Set form was not filled out as the procedure was done by staff or in facilities not familiar with routine National Joint Registry data collection. Any procedures done outside of the geographic capture of the National Joint Registry would not appear in the data and as such the stage one of two, or the stage two of two would appear to us as an incomplete two-stage revision if one of the procedures was not captured.

There are some limitations inherent to this study. We have presented a composite of all the different types of hip brands, bearings and fixations used in the primary and revision procedures, as well as combining all indications, such as septic and aseptic revisions. Additionally, using a data set with this length of follow-up combines all cases during the period of observation and the selection of different implant designs and materials, such as the introduction of highly cross-linked polyethylene, might have changed over time. Although there is evidence of the role infection plays in earlier revision,^32^ and the improved survivability of highly cross-linked polyethylene,^33^ as well as some evidence that factors such as the fixation method are correlated with risk of further revision according to patient age,^34^ we believe that the exposure to further revision itself has the greatest effect. Furthermore, subdividing the data by extra host factors would likely create small groups with resultant low power to detect any effect. Additionally, it is not yet clear how generalisable these results are to other populations around the world. However, the overall burden of revision reported by other groups, including the Swedish Hip Arthroplasty Registry and the Australian Orthopaedic Association National Joint Replacement Registry, illustrate broadly similar patterns of first revision to the data captured by the National Joint Registry.^35^, ^36^ Therefore, we do not believe there would be any substantial difference in the results. A further limitation is that up until the introduction of Minimum Data Set version 7, DAIRs were coded as a single stage revision. Therefore, some procedures in this study that are described as single stage revisions will be DAIRs.

To decide whether to undergo intervention, patients need the best possible information regarding their individual risk of needing to have further intervention in the future. We have highlighted that younger patients need to be made aware that they are at higher risk of multiple revisions. Approximately 20% of first revision will be replaced within 15 years compared with 7 years for second revision and 3 years for third revisions. Patients should also be counselled that if they do have a revisions, they are more likely to need a revision after this than they were after a primary procedure and that the period that subsequent revisions last approximately halves each time a hip is revised.

## Data sharing

The data that support the findings of this study are available from the National Joint Registry of England, Wales, Northern Ireland, the Isle of Man, and the States of Guernsey but restrictions apply to the availability of these data, which were used under license for the current study, and so are not publicly available. Data access applications can be made to the National Joint Registry Research Committee at https://www.njrcentre.org.uk/njrcentre/Research/Research-requests.

## Declaration of interests

KD is a member of the National Joint registry lot 2 contract team, tasked with doing statistical analyses for the National Joint Registry. AWB is a member of the National Joint registry lot 2 contract team, tasked with doing statistical analyses for the National Joint Registry; is the primary investigator on a grant funded by Stryker investigating the outcome of a total knee replacement manufactured by Stryker; and reports being an editor of Apley & Solomon's System of Orthopaedics and Trauma 10th Edition textbook for which he receives royalty payments. MRW's institution receives market rate payments for teaching on basic science and hip replacement that he delivers on courses organised by Heraeus and DePuy; is a member of the National Joint registry lot 2 contract team, tasked with doing statistical analyses for the National Joint Registry; and is an editor of Apley & Solomon's System of Orthopaedics and Trauma 10th Edition textbook for which he receives royalty payments. AS is a member of the National Joint registry lot 2 contract team, tasked with doing statistical analyses for the National Joint Registry; and was funded by a Medical Research Council Strategic Skills Fellowship MR/L01226X/1. SKK and JM declare no competing interests.
