# Supplementary material for: How long do revised and multiply revised hip replacements last? A retrospective observational study of the National Joint Registry
Source: Lancet Rheumatol. 2022 Jun 23;4(7):e468–79. doi: 10.1016/S2665-9913(22)00097-2 (PMC9647039; doi:10.1016/S2665-9913(22)00097-2)

THE LANCET
Rheumatology

Supplementary appendix

This appendix formed part of the original submission and has been peer reviewed. We post it as supplied by the authors.

Supplement to: Deere K, Whitehouse MR, Kunutsor SK, Sayers A, Mason J, Blom AW. How long do revised and multiply revised hip replacements last? A retrospective observational study of the National Joint Registry. *Lancet Rheumatol* 2022; **4:** e468–79.

Supplemental table 1: Kaplan-Meier estimates of cumulative 2nd revision in both study populations, stratified by age, and age and sex combined

			Time since first revision						
**Age group (years)**	**Hip type**	**N (%)**	**1 year**	**3 years**	**5 years**	**7 years**	**10 years**	**13 years**	**15 years**
All cases	Total hips	2,712 (100%)	9·32 (8·25-10·51)	15·99 (14·54-17·58)	19·91 (18·19-21·78)	22·28 (20·30-24·42)	*25·85 (23·01-28·97)*	*26·90 (23·52-30·66)*	··
	Resurfacing	457 (100%)	6·97 (4·96-9·77)	14·11 (11·10-17·86)	*17·54 (14·09-21·70)*	*20·97 (16·94-25·80)*	*22·13 (17·67-27·51)*	··	··
Females	Total hips	1,497 (55·2%)	9·38 (7·97-11·02)	16·45 (14·49-18·64)	19·48 (17·24-21·96)	21·46 (18·96-24·24)	*22·46 (19·64-25·61)*	*24·07 (20·10-28·68)*	*40·21 (23·38-62·95)*
	Resurfacing	227 (49·7%)	*6·35 (3·81-10·49)*	*15·28 (11·04-20·94)*	*19·49 (14·58-25·79)*	*22·28 (16·76-29·26)*	*24·63 (18·00-33·17)*	··	··
Female (<55)	Total hips	117 (4·3%)	*10·60 (6·16-17·91)*	*23·79 (16·50-33·58)*	*27·25 (19·13-37·92)*	*27·25 (19·13-37·92)*	*33·87 (21·25-51·12)*	*33·87 (21·25-51·12)*	*66·93 (26·74-98·05)*
	Resurfacing	66 (14·4%)	*3·03 (0·77-11·58)*	*14·00 (7·54-25·20)*	*20·85 (12·65-33·25)*	*23·40 (14·43-36·63)*	*23·40 (14·43-36·63)*	··	··
Female (55 to 64)	Total hips	284 (10·5%)	*9·94 (6·92-14·17)*	*15·50 (11·52-20·67)*	*18·22 (13·70-24·01)*	*23·53 (17·89-30·59)*	*24·74 (18·80-32·16)*	*29·76 (20·03-42·77)*	*53·17 (22·79-89·19)*
	Resurfacing	89 (19·5%)	*10·28 (5·48-18·82)*	*21·50 (14·09-32·01)*	*24·39 (16·41-35·33)*	*28·65 (19·57-40·74)*	*33·41 (21·99-48·61)*	··	··
Female (65 to 74)	Total hips	560 (20·6%)	10·71 (8·36-13·66)	19·47 (16·18-23·34)	*22·55 (18·88-26·82)*	*23·64 (19·76-28·15)*	*23·64 (19·76-28·15)*	*23·64 (19·76-28·15)*	*23·64 (19·76-28·15)*
	Resurfacing	64 (14·0%)	*4·95 (1·62-14·57)*	*8·76 (3·73-19·85)*	*11·91 (5·34-25·38)*	*11·91 (5·34-25·38)*	*11·91 (5·34-25·38)*	··	··
Female (≥75)	Total hips	536 (19·8%)	7·35 (5·37-10·01)	*11·70 (8·97-15·18)*	*14·61 (11·34-18·72)*	*15·80 (12·00-20·64)*	*15·80 (12·00-20·64)*	*15·80 (12·00-20·64)*	··
	Resurfacing	8 (1·8%)	*0*	*0*	*0*	*0*	··	··	··
Males	Total hips	1,215 (44·8%)	9·24 (7·70-11·07)	15·42 (13·33-17·80)	20·49 (17·88-23·42)	*23·41 (20·32-26·88)*	*30·62 (25·31-36·75)*	*30·62 (25·31-36·75)*	*30·62 (25·31-36·75)*
	Resurfacing	230 (50·3%)	*7·57 (4·77-11·90)*	*12·84 (8·97-18·22)*	*15·43 (11·05-21·33)*	*19·43 (14·05-26·53)*	*19·43 (14·05-26·53)*	*32·86 (14·70-63·15)*	*32·86 (14·70-63·15)*
Male (<55)	Total hips	116 (4·3%)	*6·40 (3·10-12·97)*	*14·24 (8·63-22·99)*	*18·80 (11·95-28·88)*	*20·61 (13·26-31·23)*	*24·79 (15·29-38·68)*	··	··
	Resurfacing	62 (13·6%)	*4·87 (1·60-14·33)*	*13·38 (6·92-25·01)*	*15·31 (8·25-27·41)*	*18·13 (10·02-31·54)*	*18·13 (10·02-31·54)*	*38·60 (13·80-79·85)*	*38·60 (13·80-79·85)*
Male (55 to 64)	Total hips	250 (9·2%)	*9·64 (6·51-14·16)*	*17·89 (13·42-23·64)*	*24·02 (18·56-30·77)*	*26·62 (20·45-34·22)*	*35·56 (25·58-47·98)*	*35·56 (25·58-47·98)*	*35·56 (25·58-47·98)*
	Resurfacing	85 (18·6%)	*7·19 (3·30-15·32)*	*9·95 (5·09-18·96)*	*13·20 (7·26-23·33)*	*20·25 (11·90-33·25)*	*20·25 (11·90-33·25)*	··	··
Male (65 to 74)	Total hips	439 (16·2%)	10·68 (8·08-14·06)	*17·16 (13·69-21·39)*	*22·11 (17·82-27·25)*	*26·01 (20·90-32·10)*	*34·89 (26·11-45·59)*	*34·89 (26·11-45·59)*	··
	Resurfacing	63 (13·8%)	*13·14 (6·79-24·59)*	*17·49 (9·72-30·34)*	*17·49 (9·72-30·34)*	*17·49 (9·72-30·34)*	*17·49 (9·72-30·34)*	··	··
Male (≥75)	Total hips	410 (15·1%)	8·14 (5·78-11·38)	*12·00 (8·89-16·09)*	*16·34 (12·26-21·60)*	*18·54 (13·19-25·72)*	*18·54 (13·19-25·72)*	··	··
	Resurfacing	20 (4·4%)	*0*	*7·69 (1·12-43·36)*	*23·08 (5·75-68·75)*	*23·08 (5·75-68·75)*	··	··	··

Note: Blue italics represent estimates where the number at risk has fallen below 250 cases· Blank cell indicates that the number at risk has fallen below 10, thus estimates have been removed as they would be highly unreliable.

Supplemental table 2: Number and percentage of revision episodes by indication for revision, in the total hip population

Indication for revision	1st revisions (N=25082) N (%)	2nd revisions (N=2712) N (%)	3rd revisions (N=453) N (%)
Aseptic loosening	6306 (25·1)	559 (20·6)	81 (17·9)
Dislocation / Subluxation	4753 (18·9)	706 (26·0)	111 (24·5)
Infection	4055 (16·2)	900 (33·2)	204 (45·0)
Periprosthetic fracture	3739 (14·9)	228 (8·4)	37 (8·2)
Adverse reaction to particulate debris	3323 (13·2)	146 (5·4)	14 (3·1)
Pain	2935 (11·7)	231 (8·5)	33 (7·3)
Other indication	1886 (7·5)	172 (6·3)	30 (6·6)
Malalignment	1781 (7·1)	136 (5·0)	12 (2·6)
Implant wear	1535 (6·1)	125 (4·6)	20 (4·4)
Lysis	1383 (5·5)	87 (3·2)	20 (4·4)
Implant fracture	789 (3·1)	76 (2·8)	12 (2·6)
Head-socket size mismatch	176 (0·7)	10 (0·4)	2 (0·4)

NB: Please note that, as several indications for revision can be stated, the indications are not mutually exclusive and therefore column percentages may not add up to 100%.

Supplemental table 3: Number and percentage of revision episodes by indication for revision, in the resurfacing hip population

Indication for revision	1st revisions (N=3928) N (%)	2nd revisions (N=457) N (%)	3rd revisions (N=79) N (%)
Adverse reaction to particulate debris	1421 (36·2)	110 (24·1)	5 (6·3)
Pain	1149 (29·3)	100 (21·9)	10 (12·7)
Aseptic loosening	812 (20·7)	111 (24·3)	17 (21·5)
Other indication	614 (15·6)	50 (10·9)	1 (1·3)
Periprosthetic fracture	410 (10·4)	27 (5·9)	4 (5·1)
Lysis	329 (8·4)	33 (7·2)	4 (5·1)
Malalignment	221 (5·6)	24 (5·3)	3 (3·8)
Infection	181 (4·6)	70 (15·3)	29 (36·7)
Implant wear	100 (2·5)	19 (4·2)	6 (7·6)
Dislocation / Subluxation	95 (2·4)	63 (13·8)	18 (22·8)
Implant fracture	90 (2·3)	9 (2·0)	2 (2·5)
Head-socket size mismatch	25 (0·6)	3 (0·7)	1 (1·3)

NB: Please note that, as several indications for revision can be stated, the indications are not mutually exclusive and therefore column percentages may not add up to 100%.

Supplemental figure 1: Revision schematic describing patient pathway

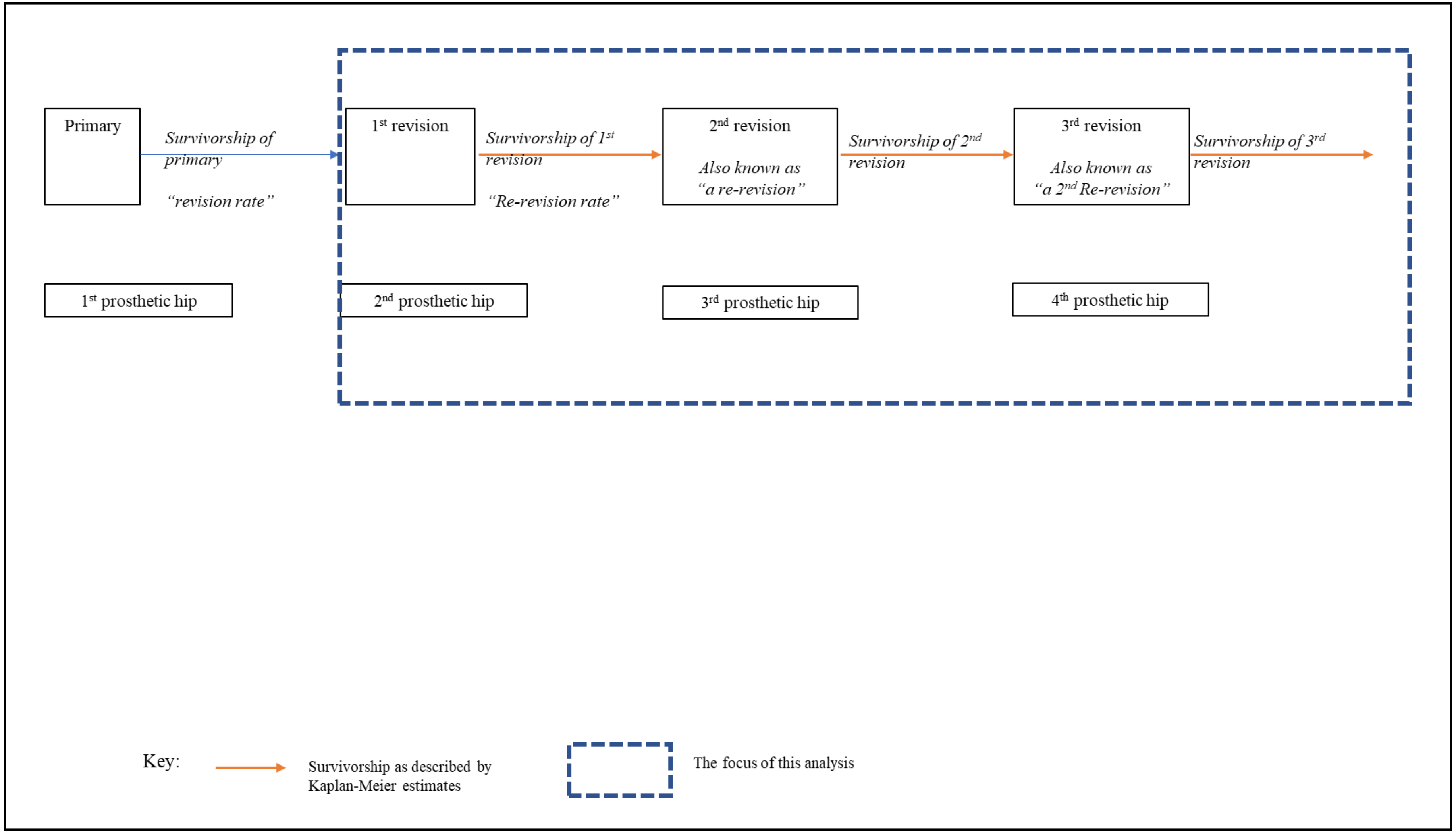

Supplement: Supplementary appendix [file mmc1.pdf]
